# Linking changes in macular thickness post cataract surgery with post-operative visual acuity

**DOI:** 10.6026/973206300211362

**Published:** 2025-06-30

**Authors:** Ramachandra Himateja C., Rashmi G.

**Affiliations:** 1 Department of Ophthalmology, Sri Devaraj URS Medical college, Tamaka, Kolar, Karnataka, India

**Keywords:** Cataract surgery, macular thickness, cystoid macular edema

## Abstract

Cataract surgery is the most commonly performed surgery, with a 90% success rate in restoring vision; however, Cystoid Macular Edema (CME) is a frequent cause of reduced vision post-operatively. This study evaluated changes in macular thickness and its correlation with post-operative visual acuity following Small Incision Cataract Surgery in 100 patients who underwent pre and post-operative macular OCT. Mean macular thickness increased from 205.14 ± 35.65 µm pre-operatively to 254.28 ± 26.68 µm at one week post-operatively, then decreased to 224.53 ± 26.73 µm by the fourth week. Visual acuity improved in 70% of patients to better than 6/9 at one week and in 96% by the fourth week post-surgery. Post-operative visual decline is often due to transient macular edema, which typically resolves without significant long-term impact on vision.

## Background:

The word cataract is derived from the Latin word "Cataracta." Cataract is the opacification of the lens and its capsule. Accumulation or improper folding of the crystalline protein causes cataracts. Cataract is classified as congenital, age-related, sub-capsular, cortical and nuclear sclerotic cataract [1, 2]. The surgical management includes extra capsular cataract extraction, small incision cataract surgery (SICS), phacoemulsification and laser surgeries. Cataract surgery involves the removal of the cataractous lens and the implantation of an intra-ocular lens [3, 4]. The SICS uses a small incision and is self-healing without sutures. SICS has the advantage of faster recovery and fewer post-operative complications like cystoid macular edema, iritis, *etc.* [4, 5]. Cystoid macular edema (CME) is an important complication occurring post cataract surgeries. It is the combination of macular thickening and fluid accumulation in the macula or central retina due to disruption in blood-retinal barrier. It has the ability to alter visual acuity and macular thickness. The visual impairment occurs between four- and twelve weeks post-surgery [6]. Macular thickness can be measured using a diagnostic modality called optical coherence tomography (OCT). The OCT is a non-invasive imaging tool that captures high-resolution, cross-sectional images. The OCT is based on the low-coherence interferometry principle. OCT is essential in diagnosing ocular pathologies [7, 8]. The alterations of the macular thickness and visual acuity in the CME and their correlation after the SICS have to be seen in detail for better evaluation. Therefore, it is of interest to measure the macular thickness and correlation between macular thickness and visual acuity after an uneventful small incision cataract surgery with posterior chamber intra-ocular lens implantation using OCT.

## Materials and Methods:

This prospective hospital-based study was conducted on 100 cataract patients with immature cataract above 18 years of age undergoing manual Small Incision Cataract Surgery in the department of Ophthalmology at a tertiary care centre from May 2023 to November 2024 after obtaining approval from the Central Ethics Committee and written informed consent from all patients. Cases with incomplete follow-ups, mature cataracts, complicated cataracts, co-existing ocular pathologies, previous ocular surgeries, pre-existing macular pathology like ARMD, macular edema, intra-operative complication and advanced ocular comorbidities significantly affecting visual outcomes were excluded. Pre-operative data included demographic data, visual acuity by Snellen's chart for distant vision, (converted to logMAR), near vision, slit lamp bio microscopy. Fundus examination by + 90D/ + 78D lens assisted slit lamp bio microscopy and indirect ophthalmoscopy. OCT was performed preoperatively and post-operatively at 1 week and 4 weeks & the Central subfield macular thickness (mean 188.80 ±27.64 µm) and Total macular volume (6.79 ± 0.392) were measured. The standard preoperative regime included pupillary dilatation using topical tropicamide (0.8%w/v) and phenylephrine (5%w/v). Routine small incision cataract surgery was performed under peribulbar anaesthesia. Preoperative and postoperative visual acuity, macular thickness was noted. Cases with intraoperative and postoperative complications were excluded.

## Statistical analysis:

Data was entered in MS excel and analysed by SPSS v27.0. Mean (SD) and median (IQR) was calculated for continuous variables. Categorical variables were expressed in frequencies and percentage. Spearman correlation was calculated between post-op VA and the macular thickness and volume, with p value below 0.05 taken as significant.

## Results:

A total of 100 participants 49 (49%) male and 51 (51%) females were included in this study. The mean age of the participants is 63.09 +8.62years (Table 1 - see PDF). After one week, 70% of patients had visual acuity of 6/9 (logMAR: 0.2) or better, which improved to 96% by the 4th week (Figure 1 - see PDF) irrespective of changes in the macular thickness. The mean macular thickness pre-operatively was 205.14 ± 35.65 µm. Postoperatively, macular thickness was 254.28 ± 26.68 µm after one week and by 4th week postoperatively macular thickness decreased to 224.53 ± 26.73 µm (Figure 2 - see PDF). The total macular volume pre-operatively was 8.43 ± 1.2 cm3. Postoperatively, macular volume was 9.3 ± 0.98 cm3 after one week and by 4th week postoperatively macular volume decreased to 8.94 ± 0.77 cm3 (Table 2 - see PDF) (Figure 3 - see PDF).No significant correlation was found between VA and macular thickness, volume in post-op at 7 days (Table 3 - see PDF) (Figures 4 - see PDF & 5 - see PDF). No significant correlation was found between VA and macular thickness, volume in post-op at 28 days (Table 4 - see PDF) (Figures 6 - see PDF & 7 - see PDF).

## Discussion:

Cataract is one of the most common pathology associated with the eye and often, the visual acuity worsens with time due to progression of cataract. In our study, patients underwent SICS. This study measured the macular thickness and its correlation with visual acuity after the small incision cataract surgery with posterior chamber intra-ocular lens implantation using OCT. Although changes in macular thickness were noted in the majority of cases after cataract surgery, no correlation was identified between these changes and visual acuity. In our study, the mean pre-op VA of the patients was 1.34 logMAR. The mean visual acuity on postoperative day 7 of the patients was 0.22 logMAR. The mean value of visual acuity on the postoperative day 28 of the patients was 0.07 logMAR, *i.e.*, the logMAR value decreased after surgery, indicating an improvement in visual acuity (a lower logMAR score corresponds to better vision). When visual acuity was converted to Snellen chart decimal values (Decimal BCVA=10-logMAR), the values showed an increase over time, *i.e.*, 0.04 on pre-op, 0.6 on post-op day 7 and 0.85 on post-op day 28. Parajuli *et al.* [9] documented that the mean pre-operative VA improved following the procedure, *i.e.*, log MAR value decreased from 0.59 to 0.19, which is similar to our study. Savarkar *et al.* [10] conducted a similar study on 95 patients who underwent uneventful cataract surgery. Patients were divided into two groups. Group S (n = 45) included 45 patients who underwent small incision cataract surgery (SICS), while Group P (n = 50) comprised patients who underwent uneventful phacoemulsification. It was documented that 98% of patients in Group P and 95.5% of patients in Group S achieved a post-operative best-corrected visual acuity (BCVA) of 6/9 by 6th week. The pattern of improvement and the post-operative value of visual acuity were comparable in the studies. Similar to our study, Dabas *et al.* [11] documented an increase in VA as per Snellen's chart from preoperative (0.05) to postoperatively in the 1st week (0.65), 6th week (0.66) and 12th week (0.67). Deepika *et al.* [12] observed that the preoperative VA as per Snellen's chart values increased from 0.06 to 0.66 at week one and 0.68 at week six. The increasing pattern of VA following the SICS was comparable in both studies. However, in our study, the assessment of VA was conducted only at two post-operative points, *i.e.*, week one and four, but in the comparison study, it was done at three points till week 12.

The enhancement in visual acuity may be attributed to the removal of the opaque cataractous lens and its replacement with a clear intraocular lens (IOL), which enables proper focusing of light onto the retina. Mean pre-operative MT and TMV of the study patients was 205.14 and 8.43 cucm. Similarly, the mean value of post-operative day 7 MT and TMV were 254.28µm and 9.3 cucm. The mean value of post-operative at day 28 of MT and TMV were 224.53 µm and 8.94 cucm. The macular thickness increased post-SICS at one week and declined at 4 weeks. The pattern was similar in TMV. Dabas *et al.* [11] documented that preoperatively, the average macular thickness was 217.03, which significantly elevated in the 1st week (221.6 µm) and 6th week (224.6 µm) and reduced in the 12th week (219.2 µm) (p<0.0001). In comparison, the MT at week one was higher in our study (254 vs 221). The pattern of increase in week one and later decline of MT was similar to our study results. Similarly, Parajuli *et al.* [9] study showed that the MT increased from 208µm in the preoperative period to 255µm in week one and declined to 246µm in week six. The increase in MT and TMV in the early postoperative phase can be attributed to subclinical inflammation, which is a physiological response to surgical trauma. The inflammation (mediators such as prostaglandins and cytokines) may lead to disruption of the blood-retinal barrier and accumulation of fluid in the macular layers, detectable by OCT. Importantly, the resolution of this thickening by the fourth postoperative week in most patients, as seen in our study, suggests that these changes are often transient and self-limiting. The self-limiting increase in macular thickness may be attributed to the physiological resorption of fluid in the later stages. Studies documented that the use of topical NSAIDs or corticosteroids post-surgery reduces prostaglandin synthesis and promotes faster resolution of macular edema [14]. Deepika *et al.* [12] observed that the MT increased from 216 at preoperative time to 221 at week 1 and 224 at week 6. The pattern was different from our study results. The probable reasons for the difference might be inflammation or edema may have peaked earlier and resolved faster in our study patients. Secondly, there might be variations in the duration of SICS, wound construction, amount of irrigation fluid used and post-operative care. In Savarkar *et al.* [10], the central subfield macular thickness (CSMT) in Group P measured 212.78 ± 17.83 µm preoperatively and 213.56 ± 24.24 µm postoperatively at 6 weeks, showing no significant change. In Group S, the mean preoperative CSMT was 206.80 ± 19.76 µm, comparable to that in the present study.

Postoperatively at week 6, it increased to 224.24 ± 49.57 µm, which is similar to the value reported in the present study at post-operative week 4. However, unlike the present study, the Savarkar study did not estimate the post-operative period MT values at week 1 and week 4. The previous studies hypothesized that the macular thickness increased immediately post-operatively, probably till week four and declined thereafter. However, our study did not estimate the prolonged observations of macular thickness following SICS. The immediate increase in macular thickness may be attributed to fluid accumulation in the macula, often resulting from ocular aberrations and inflammation. Furthermore, factors such as the length of the incision and underlying comorbidities could impact the duration of the macular thickness increase after surgery. Bhargava *et al.* [13] documented that the foveal thickness increased from 230 in pre-operative period to 235 in week one post-operation, further increased to 239 in week four and declined in week 12 to 233. The pattern was similar to our study. In our study, although a positive correlation was observed between macular thickness and visual acuity (VA), this correlation was not statistically significant at 7 days post-operation. Similarly, no significant correlation was found between VA and total macular volume. A similar lack of significant correlation was observed on the 28th postoperative day. This was because there was a significant increase in VA post-surgery in both day 7 and 28, while the macular thickness increased and declined over the postoperative period.

In a similar study conducted by Bhargava *et al.* [13] there was no correlation between the visual acuity and macular thickness. These findings suggest that routine short-term increase in macular thickness following SICS may not necessarily translate into poorer visual outcomes and do not require aggressive intervention unless clinically indicated (*e.g.*, cystoid macular edema or visual complaints). Postoperative monitoring should continue to include OCT in selected high-risk cases (*e.g.*, diabetics and intra-operative complications), but in uncomplicated cases, visual acuity remains a more immediate and practical indicator of recovery. Clinicians should also recognize that functional visual improvement can precede complete anatomical resolution and short-term macular thickening may be a normal, self-limiting physiological response. The absence of a strong correlation between MT and VA could be due to pre-existing ocular factors such as lens opacity severity, undiagnosed retinal pathology, or early diabetic changes, which may not have been apparent preoperatively. Stratification of patients based on comorbid conditions could yield more detailed insights [11]. Despite the anatomical changes; the consistent improvement in best corrected visual acuity post-operatively suggests that mild to moderate increases in MT may not adversely impact functional visual outcomes in the short term. The transient rise in macular thickness may result from natural fluid resorption over time. Studies show that postoperative use of topical NSAIDs or steroids inhibits prostaglandin synthesis, aiding quicker resolution of macular edema [14]. This has important clinical implications, particularly in resource-limited settings where access to OCT may be restricted.

## Limitations:

[1] In our study, patients were followed up only until four weeks after the SICS procedure. However, long-term follow-up and an analysis of changes in macular thickness beyond this period were not conducted.

[2] This study focused on the correlation between macular thickness and visual acuity following SICS. As a result, the generalizability of the findings regarding changes in visual acuity and macular thickness to other types of ophthalmic surgeries is limited.

[3] Patients with immature cataracts aged 18 years and older were included in the study, so the findings may not be applicable to individuals with mature cataracts.

## Conclusion:

Visual acuity improved after SICS in both the first and fourth post-operative weeks, with a transient increase in macular thickness and volume noted early on. However, no statistically significant correlation was found between visual acuity and macular parameters, indicating that short-term anatomical changes may not predict visual outcomes. OCT is valuable for high-risk or symptomatic patients, but routine use in uncomplicated cases may be unnecessary; longer-term studies are needed for deeper insight into structural-functional recovery.

## References

[1] Nizami A.A (2024). Cataract..

[2] Shiels A, Hejtmancik JF (2019). Annu Rev Vis Sci..

[3] Davis G (2016). Mo Med..

[4] Moshirfar M (2025). Cataract surgery..

[5] Gogate P.M (2009). Indian J Ophthalmol..

[6] Iuliano L (2025). Retina..

[7] Huang D (1991). Science..

[8] Nicholas S (2006). Clin Exp Ophthalmol..

[9] Parajuli S (2023). Nepal J Ophthalmol..

[10] Savarkar M (2022). Med J DY Patil Vidyapeeth..

[11] Dabas G (2022). Indian J Ophthalmol..

[12] Deepika N (2024). African Journal of Biological Sciences..

[13] Bhargava S, Devendra J (2021). IOSR Journal of Dental and Medical Sciences (IOSR-JDMS)..

[14] Wittpenn J.R (2008). Am J Ophthalmol..

